# Cost-effectiveness analysis of adding durvalumab to chemotherapy as first-line treatment for advanced biliary tract cancer based on the TOPAZ-1 trial

**DOI:** 10.1186/s12962-023-00429-9

**Published:** 2023-03-01

**Authors:** Qiuling Zhao, Ruixiang Xie, Wanfu Zhong, Wenbin Liu, Ting Chen, Xiuliang Qiu, Lin Yang

**Affiliations:** 1 Department of Pharmacy, Clinical Oncology School of Fujian Medical University, Fujian Cancer Hospital, Fuzhou, China; 2 Department of Pharmacy, Fujian University of Traditional Chinese Medicine, Fuzhou, China

**Keywords:** Durvalumab, PD-L1, Biliary tract cancer, First-line treatment, Cost-effectiveness analysis

## Abstract

**Background:**

Durvalumab plus gemcitabine and cisplatin has a significant clinical benefit for advanced biliary tract cancer (BTC). However, the high price of durvalumab warrants an exploration of the economics.

**Objective:**

To investigate the cost-effectiveness of adding durvalumab to gemcitabine and cisplatin compared with gemcitabine and cisplatin in first-line therapy of advanced BTC from the perspective of the Chinese healthcare system.

**Methods:**

According to the TOPAZ-1 trial, a three-state Markov model was built by the TreeAge Pro 2022 software. The total costs and quality-adjusted life years (QALYs) were estimated, and the incremental cost-effectiveness ratio (ICER) was used as the evaluation index. The triple 2021 Chinese per capita gross domestic product (GDP) of $37,663.26/QALY was used as the willingness-to-pay (WTP) threshold. Outputs were analyzed for two scenarios with and without a durvalumab drug charity assistance policy. In the scenario analysis, the base-case model was run multiple times with different prices of durvalumab to determine the effect on the ICER. Moreover, the robustness of the model was tested through sensitivity analyses.

**Results:**

Compared with chemotherapy alone, durvalumab plus chemotherapy resulted in an additional 0.12 QALY and an incremental cost of $18,555.19, the ICER was $159,644.70/QALY under the situation of charity assistance, and the ICER was $696,571.11/QALY without charity assistance, both exceeding the WTP threshold in China. The scenario analysis demonstrated that when the price of durvalumab fell by more than 94.2% to less than $0.33/mg, durvalumab plus chemotherapy will be more economical compared with chemotherapy alone under the situation of no charity assistance. One-way sensitivity analyses suggested that the cost of durvalumab had the greatest influence on the ICERs, and the probabilistic sensitivity analyses demonstrated that durvalumab plus chemotherapy was impossible to be cost-effective at the WTP threshold whether the charity assistance was available or not.

**Conclusions:**

Adding durvalumab to gemcitabine and cisplatin was not cost-effective for advanced BTC regardless of receiving and not receiving charitable assistance.

## Introduction

Biliary tract cancer (BTC) is a group of aggressively malignant tumors with high heterogeneity and poor prognosis, including intrahepatic cholangiocarcinoma cancer (ICC), extrahepatic cholangiocarcinoma cancer (ECC), gallbladder cancer (GBC) and ampullary cancer [1]. Most of the BTC cases are locally unresectable or metastatic at the time of diagnosis [2]. Chemotherapy stays the cornerstone of advanced BTC therapy, and gemcitabine combined with cisplatin remains the category one recommendation as first-line treatment for advanced BTC [3], but the vast majority of patients become inevitably drug resistant and have tumor recurrence after chemotherapy. The median survival is only 11.7 months [4], and novel active treatment strategies are urgently needed to improve the outcomes.

Immune checkpoint inhibitors (ICIs) targeting the programmed death receptor 1 (PD-1) and programmed death ligand 1 (PD-L1) pathways have demonstrated clinical activity in cancer, including in the first-line treatments. The evidence showed that BTC is an immunogenic cancer [5]. Recently, durvalumab, a selective, high-affinity, human IgG1 monoclonal antibody, has shown significant clinical efficacy for the treatment of several solid tumors [6, 7]. TOPAZ-1 (NCT03875235), an open-label, randomized, phase 3 clinical trial, indicated that durvalumab plus chemotherapy greatly improved survival among patients with advanced BTC [8], with the median progression-free survival (PFS) and overall survival (OS) of 7.2 months and 12.8 months, respectively. This latest result confirmed the benefits of immunotherapy combined with chemotherapy. Building on the promising activity, durvalumab plus gemcitabine and cisplatin has been recommended as Grade I first-line therapy by the National Comprehensive Cancer Network (NCCN) Guidelines of Hepatobiliary Cancers and Chinese Society of Clinical Oncology (CSCO) Guidelines of BTC [9, 10]. Moreover, durvalumab was approved as advanced BTC immunotherapy in 2022 by the FDA [11]. Although this indication has not yet been approved in China, the available evidence suggests that this protocol may be a better choice for the treatment of advanced BTC, and it is believed that in the near future, this protocol will be approved in China for the indication of advanced BTC.

The TOPAZ-1 study demonstrated a survival advantage and better tolerability for durvalumab, but durvalumab is dramatically expensive for patients. Therefore, we aimed to evaluate the cost-effectiveness of durvalumab plus chemotherapy for advanced BTC from the perspective of the Chinese healthcare system and provide a reference for optimizing the allocation of limited medical resources for clinicians and decision-makers.

## Methods

### Patients and treatments

Clinical information was derived from the randomized, double blind, global, phase 3 trial (TOPAZ-1; ClinicialTrials.gov number, NCT03875235). The inclusion criteria were as follows: age ≥ 18 years; histologically confirmed unresectable or metastatic adenocarcinoma BTC (including ICC, ECC, GBC); no previous treatment or recurrence 6 months after radical surgery or adjuvant therapy; no previous immunotherapy; Eastern Cooperative Oncology Group performance status of 0 or 1; and one or more measurable lesions per Response Evaluation Criteria in Solid Tumors version 1.1. According to the TOPAZ-1 trial, 685 patients were randomly assigned in a 1:1 ratio to receive durvalumab plus chemotherapy or placebo plus chemotherapy on a 21-day cycle for up to eight cycles. Patients received durvalumab (1500 mg) or placebo on day 1 of each cycle, in combination with gemcitabine (1000 mg/m^2^) and cisplatin (25 mg/m^2^)which were administered on days 1 and 8 each cycle. This was followed by maintenance therapy with durvalumab or placebo every 4 weeks until disease progression or unacceptable toxicity. It was assumed that patients with disease progression or intolerable toxicity in the two treatment groups would be transferred to second-line treatment, including chemotherapy, targeted therapy, ICIs and supportive care. The proportion of each second-line scheme was derived from the clinical data of the TOPAZ-1 trial. However, there were no specific subsequent therapeutic drugs in the trial, therefore, according to the NCCN and CSCO guidelines of BTC, FOLFOX (fluorouracil, leucovorin, oxaliplatin) was assumed to be the cytotoxic drug regimen as subsequent therapy based on the Grade I second-line recommended status.

### Model structure

**Fig. 1:**
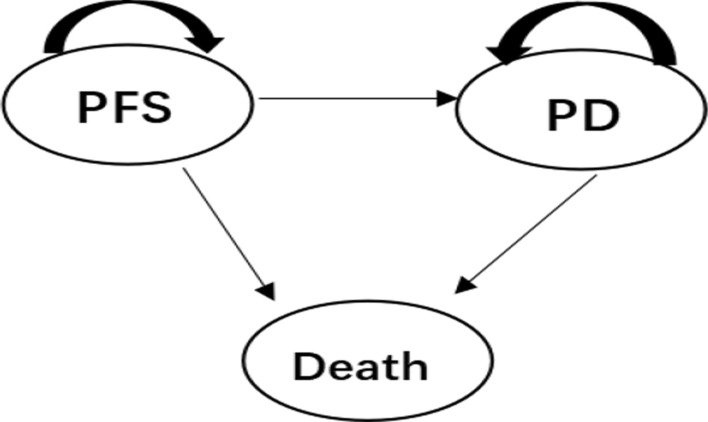
Markov model structure

Based on the TOPAZ-1 trial, a Markov model was constructed with TreeAge Pro 2022 software (Fig. 1). The model consisted of three mutually exclusive health states: PFS, progressive disease (PD) and death. The model cycle represented 21 days in keeping with the treatment schedule. Referring to the Guidelines for the Evaluation of Chinese Pharmacoeconomics (2019), a 5% discount rate was used to discount costs and health outcomes[12]. Total cost, quality-adjusted life years (QALYs) and incremental cost‒effectiveness ratio (ICER) which indicates the incremental cost of each additional QALY were the primary evaluation endpoints of the model. According to the guideline of World Health Organization, the triple 2021 Chinese per capita gross domestic product (GDP) of $37,663.26/QALY was used as the willingness-to-pay (WTP) threshold.

### Transition probabilities

OS and PFS curves data were extracted from the TOPAZ-1 study using the GetData Graph Digitizer software (version 2.25). Individual patient data of the Kaplan-Meier(K-M) curves were reconstructed from the extracted data, and the Weibull distribution was used for fit and extrapolation. The shape parameter (γ) and scale parameter (λ) were calculated using R software (version 4.2.1), and the parameters are shown in Table 1. The transition probability (p = 1 – e^–rt^) was calculated according to the DEALE principle[13]. We assumed that the probability of transition without disease progression is the natural mortality of the Chinese population in 2021.

**Table 1:** Weibull distribution parameters

Group	λ	γ	AIC (Weibull)	AIC (Exponential)
OS (durvalumab plus chemotherapy)	0.0156467	1.2807604	− 1549.654	− 1028.268
OS (chemotherapy alone)	0.009309	1.5330827	− 2404.491	− 927.0487
PFS (durvalumab plus chemotherapy)	0.029721	1.397684	− 1041.413	− 731.1559
PFS (chemotherapy alone)	0.0167583	1.754329	− 1559.848	− 709.4789

*PFS* progression-free survival, *OS* overall survival

### Costs and utilities

Only direct medical costs were estimated in the model, including the costs of drugs follow-up, adverse events (AEs) treatment and best supportive care. The prices of drugs were acquired from the Chinese health industry data center (https://www.yaozh.com). The charitable assistance project for durvalumab has been implemented in extensive stage small cell lung cancer (ES-SCLC) treatment as follows: first, the manufacturers donate durvalumab to patients after paying one cycle of treatment, and then pay for two cycles of treatment of durvalumab for free until the disease progresses. We assumed that the assistance approach was also adopted after durvalumab was approved for advanced BTC in China and analyzed the ICERs in the groups receiving and not receiving charitable assistance. We carried out a scenario analysis in this study to improve the drug affordability and explore the economic impact of this possible context in the future. Grade 3–4 AEs with a frequency of > 5% were incorporated in the model, and the costs related to AEs were equal to the incidence of 3–4 serious AEs multiplied by the handling cost per event.

In this study, we used the average body weight of 65 kg and the average body surface area of 1.72 m^2^ [14]. All costs were converted into US $2021 at the exchange rate of US $1 = 6.45 RMB.

Since the TOPAZ-1 study didn’t provide utility data, health utility values of PFS and PD were derived from published literature of 0.9 and 0.4, respectively [15].

### Scenario analysis

We conducted a scenario analysis in which the base-case model was run multiple times with different prices of durvalumab to determine the influence on the ICER when the charity assistance was not available.

### Sensitivity analyses

In one-way sensitivity analyses, variables varied over the plausible ranges of each variable, which were estimated by the 95% confidence interval (CI) or ± 25% of base case values [16]. The results were displayed by the tornado diagram. The cost details are shown in Table 2. The probabilistic sensitivity analysis (PSA) was performed by running 1000 Monte Carlo simulations. The Gamma distribution was selected for the cost parameters, and the Beta distribution was adopted for the utility value parameters. The results of the PSA are shown by the cost-effectiveness acceptability curves and scatter plots.

**Table 2:** Cost and utility parameters

Variable	Base-case value	Lower value	Higher value	Distribution	Source
*Drug costs*					
Durvalumab (1 mg)	5.61	4.21	7.01	Gamma	[17]
Gemcitabine (1 g)	52.43	39.32	65.54	Gamma	[17]
Cisplatin (30 mg)	3.84	2.88	4.80	Gamma	[17]
Pembrolizumab (100 mg)	2777.98	2083.49	3472.48	Gamma	[17]
Dabrafenib (75 mg*120)	1718.70	1289.03	2148.37	Gamma	[17]
Trametinib (2 mg*30)	1718.60	1288.95	2148.25	Gamma	[17]
Paclitaxel for injection (100 mg)	108.22	81.16	135.27	Gamma	[17]
Calcium folinate injection (100 mg)	3.88	2.91	4.85	Gamma	[17]
Regorafenib (40 mg)	26.74	20.06	33.43	Gamma	[17]
Oxaliplatin (100 mg)	78.54	58.91	98.18	Gamma	[17]
Fluorouracil (500 mg)	8.11	6.08	10.14	Gamma	[17]
*Terminal cost*	4517.85	3388.39	5647.31	Gamma	[18]
*Cost of adverse events*					
Durvalumab plus chemotherapy	603.87	452.90	754.84	Gamma	[14, 19–22]
Chemotherapy alone	591.20	443.40	739.00	Gamma	[14, 19–22]
*Costs of laboratory tests and imaging*	268.22	201.16	335.27	Gamma	Local
*Utility values*					
Progression-free survival	0.9	0.675	1.125	Beta	[15]
Progressive disease	0.4	0.3	0.5	Beta	[15]
*Discount rate*	0.05	0.0375	0.0625	Beta	[12]

## Results

### Base-case results

The base-case results are shown in Table 3. During the 10-year study period, the chemotherapy group yielded in a cost of $16,667.75, and the quality-adjusted survival was 1.68 QALYs. In the absence of charity assistance plan, the durvalumab plus chemotherapy group resulted in a cost of $97,628.84, and the quality-adjusted survival was 1.80 QALYs. Compared with the chemotherapy alone group, the QALY of durvalumab plus chemotherapy was increased by 0.12, and the incremental cost was $80,961.10, resulted in an ICER of $696,571.11/QALY. The incremental cost and ICER decreased significantly with the charity assistance plan (an incremental cost of $18,555.19 and a calculated ICER of $159,644.70/QALY). However, the ICER values are obviously exceeded the WTP threshold of $37,663.26/QALY. The key finding showed that durvalumab plus chemotherapy was not a cost-effective treatment strategy whether a charity assistance plan was available or not.

**Table 3:** Base-case results

Charity assistance	Chemotherapy alone	Durvalumab plus chemotherapy	
		Yes	No
Cost ($)	16,667.75	35,222.94	97,628.84
Incremental cost ($)		18,555.19	80,961.10
QALYs	1.68	1.80	
Incremental QALYs		0.12	
ICER($/QALY)		159,644.70	696,571.11

*QALY* quality-adjusted life-year, *ICER* incremental cost-effectiveness ratio

### Scenario analysis

The result of the scenario analysis revealed that when the price of durvalumab fell by more than 94.2% to less than $0.33/mg, durvalumab plus chemotherapy will be more cost-effectiveness than chemotherapy alone under the situation of no charity assistance.

### Sensitivity analyses

**Fig. 2:**
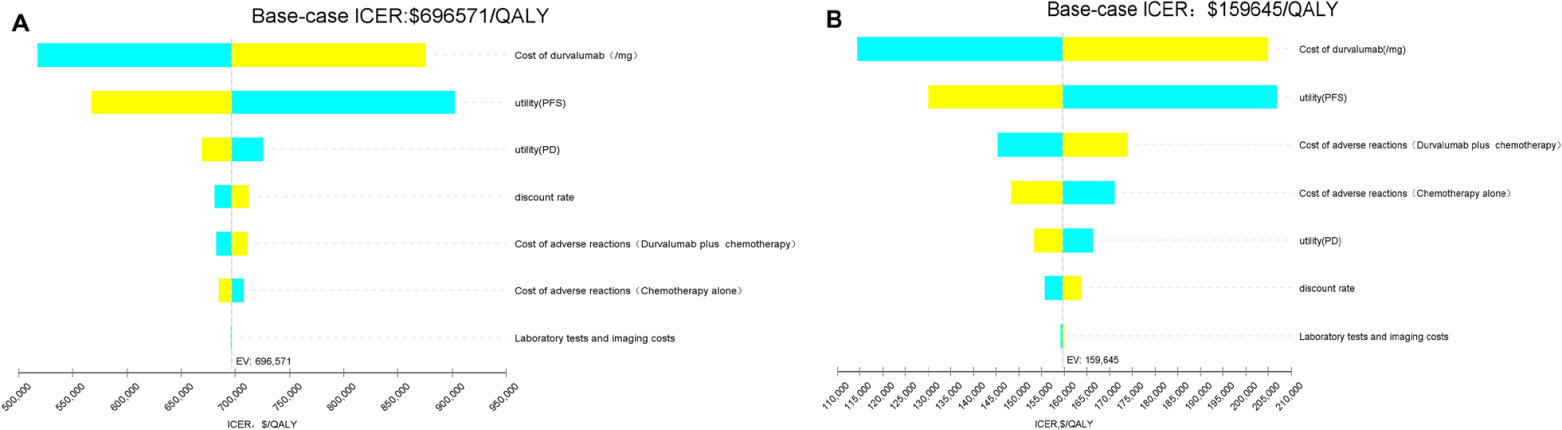
Tornado diagram of one-way sensitivity analyses **A** when the charity assistance plan was not available; **B** when the charity assistance plan was available. *PFS* progression-free survival, *PD* progressed disease, *ICER* incremental cost-effectiveness ratio

Figure 2A and B showed the results of one-way sensitivity analyses. The parameters that had the substantial impact on the ICERs were similar in the two scenarios. The cost of durvalumab had the greatest impact on the ICERs, but all of the parameters within their plausible ranges did not cause the reversal of the basic analysis results, which demonstrated the robustness of the model. In other words, no parameter causes an ICER lower than the WTP threshold, whether charity assistance plan is available or not.

**Fig. 3:**
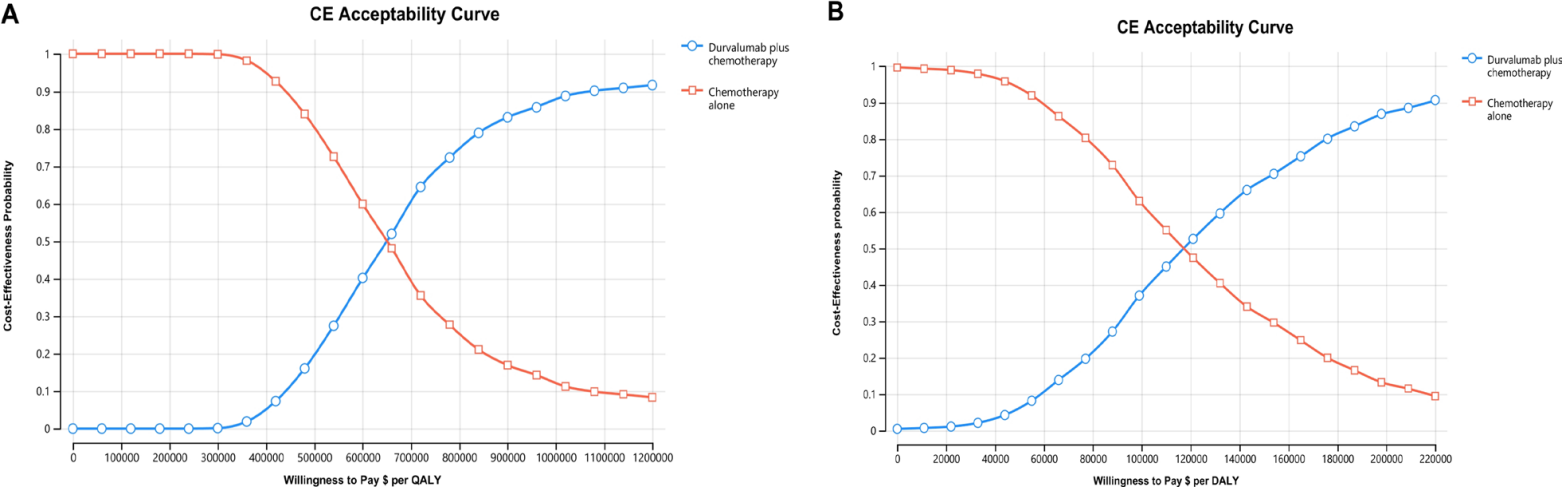
Cost-effectiveness acceptability curves **A** when the charity assistance plan was not available; **B** when the charity assistance plan was available

**Fig. 4:**
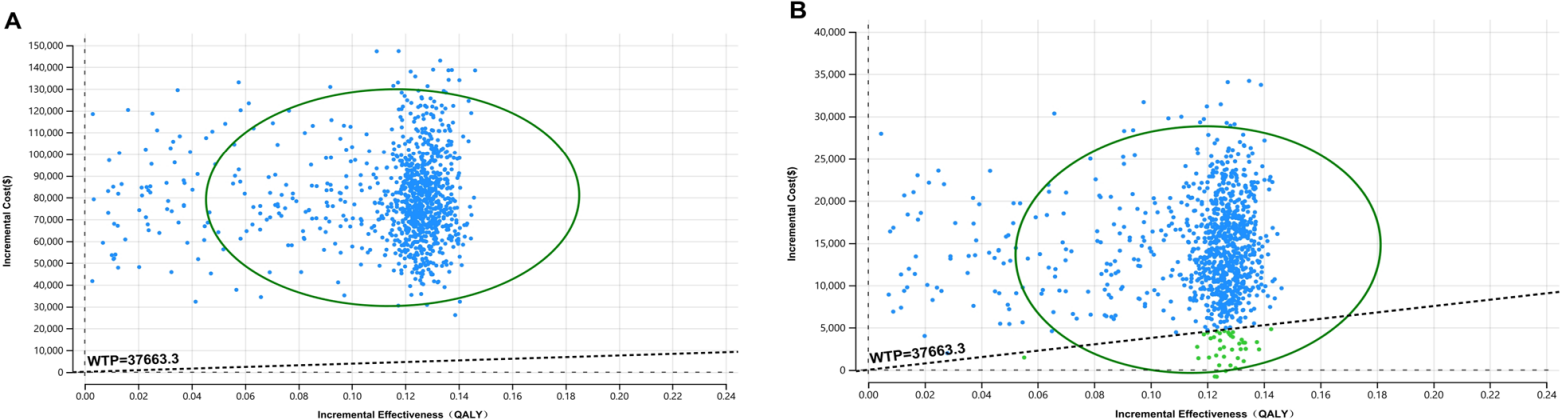
Cost-effectiveness scatter plot **A** when the charity assistance plan was not available; **B** when the charity assistance plan was available

The cost-effectiveness acceptability curves shown in Fig. 3A and B presents the results of the PSA. When the durvalumab charity assistance plan was not available, the cost-effective probability of durvalumab combined with chemotherapy compared with chemotherapy alone was 0% at the WTP threshold of $37,663.26/QALY. When the durvalumab charity assistance plan was available, the economy of durvalumab plus chemotherapy has improved but is still very low, with the cost-effectiveness acceptability of the solution at less than 10% when WTP threshold is $37,663.26/QALY. The ICER scatter points were located above the first quadrant of the coordinate axis, showing that durvalumab plus chemotherapy led to better QALY but higher costs (Fig. 4A, B).

## Discussion

Treatment for advanced BTC is developing and is no longer limited to chemotherapy [23]. Combining chemotherapy and checkpoint inhibitors may potentially take advanced BTC treatment strategies to the next level. To date, the TOPAZ-1 study is the only phase III study to demonstrate a dual benefit of immunotherapy combined with chemotherapy for PFS and OS in the treatment of advanced BTC, durvalumab hereby became a new choice for advanced BTC as a first-line treatment. Durvalumab has been approved for consolidation treatment of stage III unresectable non-small cell lung cancer (NSCLC) and the first-line treatment of extensive stage ES-SCLC in China. With the widespread use of durvalumab, the increased fiscal burden has become a problem that attracts the attention of decision-makers. Currently, there have been some cost-effectiveness studies of durvalumab in NSCLC and ES-SCLC. CRISS et al. found that in the US medical system, durvalumab consolidation therapy was cost-effective after chemotherapy and radiotherapy in patients with unresectable stage III NSCLC [24]. However, durvalumab was not cost-effective in the treatment of ES-SCLC [25]. Relevant economic evaluation of immunotherapy combined with chemotherapy as first-line treatment for advanced BTC has not been searched in other countries through literature retrieval. To the best of our knowledge, this is the first study aimed at analyzing the cost-effectiveness of durvalumab plus chemotherapy versus chemotherapy alone for the first-line treatment of advanced BTC, which has been recommended by the latest clinical guidelines, and our results have important implications for decision makers.

Based on our results, adding durvalumab to gemcitabine and cisplatin produced an ICER of $696,571.11/QALY without charity assistance plan, higher than the generally accepted WTP threshold of $37,663.26/QALY. If this charity assistance plan can also be implemented for advanced BTC, it will bring better benefits to patients than those without a charity assistance plan. We included the charity plan in the Markov model and found that the ICER was $159,644.70/QALY, which improved economic benefits. Hence, we suggest the company applying this charity plan to advanced BTC patients so that more Chinese patients can obtain better economic benefits. We also conducted a scenario analysis and showed that when the price of durvalumab is reduced by more than 94.2% to less than $0.33/mg, the ICER will be lower than the WTP threshold. Therefore we also hope that the price of durvalumab will be reduced in the future so that this effective treatment option can be applied to a wider range of patients.

One-way sensitivity analyses showed that the ICERs were most sensitive to the price of durvalumab. The total cost of durvalumab plus chemotherapy with receiving charity assistance plan is $62,405.90 more than that without receiving charity assistance plan. Therefore, we hope that drug manufacturers would implement charitable assistance programs to enhance the accessibility and economics of durvalumab. Due to the differences in GDP levels and local WTP for patients in different countries, drug manufacturers can adopt different assistance strategies globally to cope with price factors affecting the use of durvalumab in patients. China has implemented a national drug procurement negotiation mechanism to control drug costs. The prices of ICIs (such as camrelizumab, toripalimab, sintilimab and tislelizumab) have dropped significantly even more than 60%, and have been included in medical insurance. With the launch of several phase III clinical trials, an increasing number of PD-1 inhibitors will be put into use, which will provide new immunotherapy possibilities for advanced BTC patients.

There are some potential limitations in this study. First, this trial recruited 685 advanced BTC patients in global and there were only 374 Asian participants, but the results can be certainly extented to the Chinese population. If there is a subgroup analysis of the Chinese population in the near future, the results of this study can be further validated. Second, our model is dependent on the validity and generalizability of the phase III clinical trial rather than a prospective real-world study, and the subsequent treatment was based on the recommendation of NCCN and CSCO Guidelines for advanced BTC, without considering the individualization of patients in reality. Third, our model only included direct medical costs but not indirect costs, which may result in inaccurate cost estimation. However, one-way sensitivity analyses proved that these costs had little effect on the model results, except for the costs of durvalumab. This clinical trial is still ongoing, and the parameters may need to be updated in the future if new clinical data are published. Despite these limitations, the results of this study reflect the general clinical conditions of advanced BTC. These results represent an important step forward in getting a better balance between improving health outcome and saving medical expenses, and also have reference value for guiding the rational allocation of the resource-limited countries such as China.

## Conclusions

In summary, when the WTP threshold is $37,663.26/QALY, durvalumab plus gemcitabine and cisplatin is not considered to be cost-effective compared with chemotherapy alone for patients with advanced BTC whether a charitable assistance project was considered or not. Providing more favorable charity assistance plan for advanced BTC would have a higher chance to become cost-saving.
